# Predictive ability of a commercial mixed-breed genomic test for feedlot performance and carcass traits of beef × Holstein steers

**DOI:** 10.1093/jas/skaf053

**Published:** 2025-02-25

**Authors:** Bailey L Basiel, Tara L Felix, Chad D Dechow

**Affiliations:** Department of Animal Science, Pennsylvania State University, University Park, PA, USA 16802; Department of Animal Science, Pennsylvania State University, University Park, PA, USA 16802; Department of Animal Science, Pennsylvania State University, University Park, PA, USA 16802

**Keywords:** beef on dairy, carcass characteristics, feedlot performance, genotype, molecular breeding values

## Abstract

Genomic tests are marketed as a method to appraise cattle value prior to feedlot entry. We aimed to evaluate the use of a commercial, multi-breed genomic test to predict terminal production characteristics of beef × Holstein steers. Phenotypes of feedlot performance and carcass characteristics were evaluated in beef × Holstein steers (*n* = 259) sired by 8 beef breeds. Steers were genotyped with Igenity Beef (Neogen Corporation, Lansing, MI), which ranks cattle for individual traits and by selection indexes on a scale of 1 to 10. Trait rankings were converted to molecular breeding values (**MBV**) derived from genotype. Expected progeny differences (**EPD**) of each steer’s sire were accessed from their respective breed associations. U.S. Meat Animal Research Center across-breed adjustments for beef cattle were applied to growth and carcass sire EPD for all cattle except for the 11 Wagyu-sired steers (*n* = 248 steers with sire EPD). Breed-adjusted sire EPD and MBV of traits were correlated with associated phenotypes of beef × Holstein steers and phenotypes were regressed on sire EPD and MBV. Sire EPD and MBV of yearling weight (**YW**) and hot carcass weight (**HCW**) were positively associated with initial and final feedlot body weight, respectively. The MBV of average daily gain (**ADG**) was not associated with phenotypic ADG, though greater derived sire EPD of ADG was associated with greater ADG and dry matter intake (**DMI**). The MBV of residual feed intake (**RFI**) was associated with DMI (*P *= 0.02) but not RFI or gain-to-feed ratio. Each kg of RFI predicted by MBV resulted in 0.31 kg greater ADG (*P *< 0.01), suggesting that MBV of RFI in beef × Holstein steers are not independent of the rate of gain. For each kg of HCW predicted by MBV and sire EPD carcasses were 0.52 and 0.80 kg heavier (*P *< 0.01). Neither sire EPD nor MBV of ribeye area and backfat thickness were related to the corresponding phenotypes of beef × Holstein carcasses (*P *> 0.05). Both MBV and sire EPD of marbling score were strong predictors of marbling score and intramuscular fat content (*P *< 0.05). Tenderness MBV accurately predicted tenderness of the longissimus muscle of beef × Holstein progeny (*P *< 0.01). Igenity Beef Terminal Index ranking was associated with greater feedlot profitability (*P *= 0.03), but marbling MBV and sire EPD were more strongly associated with feedlot net profit (*P *< 0.01). Breed-adjusted sire EPD performed similarly to Igenity Beef MBV in predicting growth performance, carcass characteristics, and net profit of beef × Holstein steers.

## Teaser

Sire expected progeny differences perform similarly to a commercial genomic test in predicting growth performance, carcass performance, and terminal value of beef × Holstein steers.

## Introduction

In the United States, dairy farms have rapidly adopted the practice of mating a portion of their herd to beef-breed semen to add value to surplus calves (Basiel and Felix, 2022). An increasing portion of the nation’s fed cattle supply is comprised of the beef × dairy progeny resulting from such matings (Mayer et al., 2024). Currently, day-old beef × dairy calves are being sold at auction for $300 to $400 more than Holstein bull calves (USDA, 2024). However, it is unclear if the premiums paid for beef × dairy calves are maintained through the rest of the beef supply chain; reports of inconsistent musculature of live beef × dairy cattle and carcasses suggest that further valuation of beef crossbred progeny is variable (Foraker et al., 2022a).

The ranking of individual cattle by molecular breeding values (**MBV**), derived from genomic markers, has been proposed as a method for cattle feeders to determine the value of an animal prior to feedyard placement and to consider customized management strategies for cattle grouped by genetic potential (Thompson et al., 2014). Cattle farmers are interested in harnessing genomics to market feeder cattle. In fact, 56% of surveyed cattle producers indicated they were willing to pay $21 per head, on average, for genomic testing (DeLong et al., 2023). Commercial genomic tests offered for beef cattle are typically marketed for use in replacement heifer selection (Andersen et al., 2022). However, one company, Neogen, markets 3 genomic panels for use in commercial-fed cattle: Igenity Beef, Igenity Feeder, and Igenity BeefxDairy (Neogen, 2021, 2023a, 2023b).

Igenity Beef is marketed for both breeding animals and terminal cattle; the test costs $30 per animal and uses genomic information to rank animals on 17 traits and 3 selection indexes (Neogen, 2023a). Igenity Feeder only costs $15 and ranks feeder cattle on the same terminal index (TI) used by Igenity Beef (Neogen, 2021). Igenity Feeder also provides a day-on-feed index to allow for precision management of pens (Neogen, 2021). Igenity BeefxDairy is marketed specifically for use in fed beef × dairy cattle (Neogen, 2023b). The test costs $17 and ranks animals for average daily gain (**ADG**), marbling, and hot carcass weight (**HCW**) in addition to ranking them on the same TI used by Igenity Beef (Neogen, 2023b). Feeder cattle with genotypes from 1 of the 3 aforementioned Neogen tests qualify to be marketed through Igenity Branded, a program which offers premiums for feeder cattle based on genomic predictions of their performance (Courter, 2021). From 2020 to 2021, feeder cattle marketed through Igenity Branded averaged a $59 per animal premium over cattle groups without genomic predictions (Courter, 2021).

As commercial genomic tests have become available, researchers have emphasized the importance of independent validation of such panels (Van Eenennaam et al., 2007). Recently, one research group validated the use of a genomic test by comparing growth and carcass phenotypes of the progeny of commercial Angus cows to the cows’ genotype (Arisman et al., 2023). Studies that have validated MBV of terminal beef cattle with phenotypes from the genotyped animals are limited to genomic panels that are no longer commercially available and industry white papers (DeVuyst et al., 2011; Neogen, 2021, 2022).

Accurate, low-cost, genomic predictions of growth and carcass traits could be used by calf buyers to assess the value that should be afforded to beef × dairy progeny. However, the efficacy of genomic panels, designed using phenotypes from beef breeds, in predicting the performance of beef × Holstein progeny has never been investigated. Thus, the objective of this research is to validate commercial multi-breed genomic predictions for feedlot performance traits and carcass characteristics of beef × Holstein progeny. Additionally, we aim to compare the predictive ability of the genomic test to the predictive ability of breed-adjusted sire expected progeny differences (**EPD**).

## Materials and Methods

This research was approved by the Pennsylvania State University Institution Animal Care and Use Committee (Protocol # 202001343).

### Cattle procurement and management

Three groups of beef × Holstein steers (*n* = 259) were fed over 3 yr (2021, 2022, and 2023) for this study. Sire selection was previously described by Basiel et al. (2024). Briefly, the steers fed in 2021 (*n* = 31) were sourced from Pennsylvania dairy farms that mated a portion of their herd to commercially available semen from Angus or SimAngus bulls. Steers fed in 2022 (*n* = 125) and 2023 (*n* = 103) were also born on Pennsylvania dairy farms but resulted from planned matings to beef-breed sires selected by terminal EPD values. Sire breeds included Angus, Red Angus, Charolais, Hereford, Limousin, SimAngus, Simmental, and Wagyu.

Calves were managed similarly year-to-year; management is described in greater detail by Basiel et al. (2024). Single-born, male calves were picked up from the dairy within 2 wk of age and raised at a commercial wet calf growing facility through weaning. Calves were castrated at 5 wk of age and weaned at 7.5 ± 2.5 wk of age and moved to a bedded pack commercial growing facility. Steers were fed corn grain, pelleted grain, and free choice hay until they were approximately 6 ± 2 mo old; then, they were transitioned to a corn silage-based TMR diet that provided 1.36 Mcal NE_g_/kg. Steers received 2 implants containing progesterone and estradiol benzoate while housed at the commercial facility. Steers were transported to the Pennsylvania Department of Agriculture Livestock Evaluation Center (**LEC**) feedlot at 9.5 ± 2.5 mo old to be finished. There, they were transitioned to a high-energy feedlot diet and implanted with a terminal implant containing TBA and estradiol. Steer groups were fed at the LEC for 90 to 153 d. Steers were selected for slaughter in groups of approximately 30 or 60 animals. Body weight was used to create groups for slaughter, with the caveat that some cattle in the last group slaughtered each year may not have reached the target body weight but were still slaughtered due to facility limitations. Steers were transported 312 km to a commercial beef processing facility and slaughtered according to the Humane Slaughter Act.

### Phenotype collection

Detailed methods of phenotypic data collection were previously described by Basiel et al. (2024). In short, steers were weighed on 2 consecutive days following feedlot arrival; weights were averaged to determine initial body weight. Likewise, steers were weighed 2 consecutive days prior to slaughter and final body weight was the average of the 2-d weights with 2.5% subtracted to account for shrink. Feedlot ADG was calculated as the difference between final and initial body weight divided by days on feed. Daily feed intake of individual steers was monitored using the GrowSafe Feed Intake System (Model 4000E; Vytelle, LLC., Lenexa, KS) during finishing at the LEC. For this study, feedlot residual feed intake (**RFI**) was estimated in addition to the gain-to-feed ratio as a measure of feed efficiency. Feedlot RFI was estimated separately for each year on feed to account for environmental variation between years. Dry matter intake (**DMI**) was regressed on feedlot ADG and midpoint metabolic body weight. Midpoint metabolic body weight was calculated as initial body weight summed with half of the total gain over the feeding period raised to 0.75. Feedlot RFI was comprised of the residuals of the DMI model.

Following removal of the hide, head, and organs, each carcass side was weighed; weights of both carcass sides were summed and 2.5% of the total was added to account for the removal of the kidneys, heart, and pelvic fat to determine the total HCW. Three days following slaughter, trained research personnel evaluated additional carcass characteristics including ribeye area, backfat thickness, and marbling score. A 3-rib section of the longissimus muscle, from the 10th to 12th rib, was cut from each carcass and transported to the Penn State Meat Science Laboratory for Warner-Bratzler Shear Force tenderness and intramuscular fat (**IMF**) determination, as described by Basiel et al. (2024).

Net profit was calculated for individual steers by subtracting input expenses from carcass value when sold on the grid. Input expenses included feedlot purchase price, feed costs, and yardage costs. Pennsylvania cattle auction records during the week of purchase for each group of cattle were used to calculate a purchase price for steers (USD/45.4 kg) at feedlot arrival (USDA, 2021a, 2022a, 2023a). Auction records report average sale prices (USD/45.4 kg) for cattle grouped by sex and weight range; prices of steers were further averaged in groups of 22.7 kg starting at 181.4 kg e.g., the purchase price of steers that weighed between 181.4 and 204.1 kg was the average price (USD/45.4 kg) of feeder steers auctioned in that weight range in Pennsylvania during the week of purchase. The average price was then multiplied by feedlot initial body weight to determine the true purchase price of each individual steer. Feed and yardage costs were determined using as-fed individual feed intake and days on feed. Base price (USD/45.4 kg) for a UDSA Yield Grade 3, Quality Grade Choice carcass was determined using the USDA Beef Carcass Price Equivalent Index Value from the first week of September of each respective year, as follows: 2021 = $252.43, 2022 = $230.03; 2023 = $289.13 (USDA, 2021b, 2022b, 2023b). The simple average of premiums and discounts were applied to the base price of individual carcasses based on values from the National Weekly Direct Slaughter Cattle—Premiums and Discounts report from the first week of September of each respective year (USDA, 2021c, 2022c, 2023c). Premiums and discounts were applied based on carcass weight, USDA Quality Grade and quality characteristics, and USDA Yield Grade. USDA Quality Grade was determined by marbling score and USDA Yield Grade was calculated using the USDA Yield Grade Equation (USDA, 2017).

### Genomic information

Blood or tail hair with roots were sampled from all cattle to facilitate genotyping with the Igenity Beef chip (Neogen Corporation, Langsing, MI). Currently, Neogen markets 3 separate chips that provide genomic predictions related to terminal production traits of crossbred beef cattle: Igenity Beef, Igenity Feeder, and Igenity BeefxDairy (Neogen, 2021, 2023a, 2023b). As its name implies, the Igenity BeefxDairy genomic panel is marketed specifically for terminal beef × dairy progeny; however, was not commercially available for use until after the initiation of this experiment (Neogen, 2023b). The BeefxDairy genomic panel ranks cattle by ADG, carcass weight, and marbling and ranks cattle on a TI (Neogen, 2023b). Igenity Feeder provides a genomic ranking of cattle on the same TI and on a predicted days-on-feed index to aid cattle feeders with group management (Neogen, 2021). Igenity Beef was selected for this study because it provides genomic predictions for 17 traits in addition to ranking cattle from 1 to 10 on 3 indexes: a production index, a maternal index, and the same TI (Neogen, 2021, 2023a). There is no publicly available information on the reference population or genomic markers used to develop Igenity genomic tests. However, MBV associated with an animal’s ranking for a trait are reported on the same scale for Igenity BeefxDairy and Igenity Beef (Neogen, 2023a, 2023b).

The Igenity Beef panel is marketed for use in both breeding stock and terminal cattle (Neogen, 2023a). Thus, genomic predictions were available for maternal and reproductive traits that were not evaluated as they were not relevant to the terminal cattle in this study. Genomic predictions of relevant growth and carcass traits included yearling weight (**YW**), ADG, RFI, HCW, ribeye area (**REA**), fat thickness (**FAT**), marbling, and tenderness (Neogen, 2023a). Cattle are ranked from 1 to 10 for each trait with ranking linearly corresponding to continuous molecular progeny differences (**MPD**) for the respective trait (Neogen, 2023a). For example, Igenity scores of 1 and 10 for HCW corresponded to MPD of 0.0 and 46.5 kg of HCW, respectively (Neogen, 2023a). For this study, MPD were converted to MBV by doubling MPD to represent the true breeding value of the terminal steer, rather than the half of the breeding value that would be transmitted to his progeny (Table 1). Therefore, Igenity scores of 1 and 10 for HCW were converted to MBV of 0.0 and 93.0 kg of HCW, respectively (Table 1). The ranking of individual steers on the Igenity TI was also utilized in this experiment. The TI puts positive weights on the MPD of HCW (40%), REA (10%), marbling (20%), and tenderness (5%) and negative weights on FAT (−10%) and RFI (−15%; Neogen, 2023a). Breeds supported by Igenity Beef are Angus, Gelbvieh, Hereford, Limousin, Maine-Anjou, Red Angus, Shorthorn, and Simmental (Neogen, 2023a).

**Table 1.** The molecular breeding values (MBV) that correspond with Igenity Beef scores for the traits evaluated. The published molecular progeny differences (Neogen, 2023a) were converted to MBV by multiplying the values by 2.

Igenity beef score		Trait						
	YW, kg	HCW, kg	ADG, kg	RFI, kg	REA, cm^2^	FAT, cm	MARB^1^	TEND, kg
1	0.0	0.0	0.00	0.00	0.0	0.00	0.00	0.00
2	8.8	10.3	0.03	0.07	2.6	0.15	0.34	−0.09
3	17.5	20.7	0.05	0.14	5.2	0.30	0.66	−0.18
4	26.3	31.0	0.08	0.21	7.7	0.41	1.00	−0.36
5	35.1	41.4	0.11	0.28	10.3	0.56	1.34	−0.54
6	43.8	51.6	0.14	0.34	11.6	0.71	1.66	−0.54
7	52.6	62.0	0.16	0.42	14.2	0.86	2.00	−0.73
8	61.4	72.3	0.19	0.49	16.8	1.02	2.34	−0.91
9	70.1	82.6	0.22	0.55	19.4	1.17	2.66	−0.91
10	78.9	93.0	0.24	0.63	21.9	1.27	3.00	−1.09

YW, yearling weight; HCW, hot carcass weight; ADG, average daily gain; RFI, residual feed intake; REA, ribeye area; FAT, fat thickness; MARB, marbling score; TEND, tenderness; TI, terminal index.

^1^Numeric marbling scores range from 1.0 to 9.9 where a score of 4.0 = Small^00^, a score of 5.0 = Modest^00^, etc.

Paternal parentage was verified by genotype to ensure that sire EPD were correctly matched to each steer. Sire EPD were accessed from their respective breed’s herdbook; the EPD were current as of March 1, 2024. Relevant sire EPD, within-breed ranking, and EPD accuracy are available in Supplemental Table 2 of Basiel et al. (2024). The 2023 U.S. Meat Animal Research Center across-breed adjustment factors were applied to sire EPD across their respective breeds (Kuehn and Thallman, 2023). Adjustment factors are available for the growth traits of weaning weight and YW, and the carcass traits of HCW, REA, FAT, and marbling score. A breed-adjusted postweaning ADG EPD was computed by taking the difference between breed-adjusted YW EPD and breed-adjusted weaning weight EPD and dividing it by 160 d.

Simmental breed adjustments were applied to the EPD of SimAngus sires because Simmental and SimAngus EPD are derived from the same population. The SimAngus sires are registered with the American Simmental Association and still represent the genetic makeup of the Simmental breed in the United States, as purebred Simmental (≥87.5% Simmental lineage by pedigree) cattle represent less than 1/3 of the breed’s herd book (ASA, 2024). Across-breed adjustment factors are not calculated for the Wagyu breed therefore, the Wagyu × Holstein data was excluded from the sire EPD analyses, resulting in 248 phenotypes of beef × Holstein steers sired by 27 individual bulls.

### Statistical analysis

Table 2 contains descriptive statistics of the growth and carcass phenotypes of beef × Holstein progeny and lists the traits with available Igenity MBV and breed-adjusted sire EPD that were evaluated in relation to each phenotype. Breed-adjusted sire EPD for growth traits were limited to weaning weight, YW, and the derived postweaning ADG EPD. Thus, the ability of sire EPD of postweaning ADG to predict feed intake and the feed efficiency phenotypes measured was evaluated. Likewise, the ability of Igenity MBV for RFI and ADG to predict other growth traits was examined. Yearling weight was selected to compare with initial body weight because genetic evaluations of weaning weight standardize phenotypes to 205 d, the average age a beef calf is weaned from its dam (Bormann and Rolf, 2021). In contrast, the beef × Holstein steers in this study were, on average, weaned at 53 d of age. Initial feedlot body weights were measured closer to when steers were yearlings than when they were weaned, which made YW the more logical breeding value to evaluate. The ability of HCW EPD and MBV to predict final body weight was evaluated because HCW better represents steer weight after 1 yr of age.

**Table 2.** Descriptive statistics of phenotypes from beef × Holstein steers and the corresponding traits with Igenity Beef molecular breeding values (MBV) and sire expected progeny differences (EPD) traits they were correlated and modeled with

Phenotype	*n*	Mean	SD	Median	Minimum	Maximum	MBV traits	EPD traits
Initial body weight, kg	259	417	77	427	197	635	YW	YW
Final body weight, kg	259	641	62	644	424	784	HCW	HCW
Average daily gain, kg/d	259	1.72	0.21	1.71	1.10	2.31	ADG, RFI	ADG
Dry matter intake, kg/d	257	14.23	1.49	14.28	8.60	17.91	RFI	ADG
Gain-to-feed ratio	257	0.12	0.01	0.12	0.08	0.17	RFI	ADG
Residual feed intake, kg	257	0.00	1.07	0.07	−4.77	3.10	RFI	ADG
Hot carcass weight, kg	259	395	45	395	241	496	HCW	HCW
Ribeye area, cm^2^	258	84.5	7.5	84.2	65.2	106.5	REA	REA
Backfat thickness, mm	257	9.8	4.4	8.9	2.5	27.9	FAT	FAT
Marbling score^1^	258	4.60	0.90	4.70	2.00	7.20	MARB	MARB
Intramuscular fat, %	255	4.54	1.79	4.32	1.26	11.06	MARB	MARB
Warner-Brazler shear force, kg	255	4.22	1.18	4.03	1.69	9.24	TEND	—
Net profit, USD	257	327.15	293.25	284.90	−237.70	972.92	TI	—

YW, yearling weight; ADG, average daily gain; RFI, residual feed intake; HCW, hot carcass weight; REA, ribeye area; FAT, fat thickness; MARB, marbling score; TEND, tenderness; TI, terminal index.

^1^Numeric marbling scores range from 1.0 to 9.9 where a score of 4.0 = Small^00^, a score of 5.0 = Modest^00^, etc.

Breed-adjusted sire EPD were Pearson correlated with Igenity MBV of the same traits using the CORR procedure of SAS (v. 9.4, Cary, NC); sire EPD of postweaning ADG was also correlated with Igenity MBV of RFI. The relationships between phenotypic traits and breed-adjusted sire EPD or Igenity MBV were also evaluated by Pearson correlation followed by a Fisher’s z transformation. Correlations were considered different from 0 when *P* ≤ 0.05. Phenotypes were fitted by linear mixed models using the MIXED procedure of SAS. Models included the fixed effect of year fed (2021, 2022, or 2023), a fixed regression on across-breed-adjusted sire EPD or Igenity MBV of relevant traits, and the random effect of herd of origin (1 to 6). Fixed effects were considered significant in models when *P *≤ 0.05 and were considered tendencies when 0.05 < *P* ≤ 0.10. Figures were generated using the ggplot2 package in R (Wickham, 2016).

A modified dataset was created containing only the phenotypes and Igenity MBV of Angus, Red Angus, Hereford, Limousin, SimAngus, and Simmental-sired beef × Holstein progeny (*n* = 157). Charolais and Wagyu-sired progeny were removed from the data because Igenity Beef does not support the Charolais or Wagyu breed. The modified data were evaluated with the same mixed models that included Igenity MBV to determine if the unsupported sire breeds were skewing Igenity MBV.

## Results and Discussion

Pearson correlation coefficients between across-breed-adjusted sire EPD and Igenity MBV are presented in Table 3. Correlations between breed-adjusted sire EPD and Igenity MBV were moderate and positive, with the weakest relationship between EPD and MBV for HCW (*r* = 0.33) and the strongest relationships between EPD and MBV for the traits backfat thickness and marbling score (*r* = 0.80 and *r* = 0.79, respectively). The strength of the relationship between sire EPD of YW and Igenity MBV of YW (*r* = 0.39) was similar to that of sire postweaning ADG EPD with ADG MBV (*r* = 0.49) and RFI MBV (*r* = 0.39). The moderate correlations suggest that sire EPD and Igenity MBV rank a beef × Holstein steer’s genetic potential for the evaluated traits similarly. It is likely that the correlations between EPD and MBV are not stronger because sire EPD only accounts for the paternal half of the steer’s genetic potential for a trait while MBV utilizes an individual’s actual genotype.

**Table 3.** Pearson correlation coefficients between breed-adjusted expected progeny differences (EPD) of the sires of beef × Holstein steers (*n* = 248) and Igenity Beef molecular breeding values (MBV) derived from their own genotypes. Sire EPD and MBV of Wagyu-sired steers are excluded because across-breed EPD adjustments are unavailable for the breed.

Sire EPD	Igenity MBV	Pearson correlation coefficient	*P*-value
YW	YW	0.39	<0.001
ADG	ADG	0.49	<0.001
ADG	RFI	0.39	<0.001
HCW	HCW	0.33	<0.001
REA	REA	0.59	<0.001
FAT	FAT	0.80	<0.001
MARB	MARB	0.79	<0.001

YW, yearling weight; ADG, average daily gain; RFI, residual feed intake; HCW, hot carcass weight; REA, ribeye area; FAT, fat thickness; MARB, marbling score.

Sire EPD and correlation coefficients between phenotypes and breed-adjusted sire EPD (Table 4) and between phenotypes and Igenity MBV (Table 5) were weak across comparisons. However, genetic variation typically explains less than half of the phenotypic variation around a trait, even when heritability is high (Uterera and Van Vleck, 2004; Ladeira et al, 2021). Furthermore, the correlations reflect phenotypes that are not adjusted for external factors such as farm of origin. Thus, we consider the direction of the correlations informative regardless of the correlation magnitude; correlation strength will be discussed in relation to the strength of the correlation of a specific phenotype with its respective Igenity MBV in contrast with the strength of the correlation of the same phenotype with its respective sire EPD.

**Table 4.** Pearson correlation coefficients between phenotypes of beef × Holstein steers (*n* = 248) and breed-adjusted sire expected progeny differences (EPD) of relevant traits; data exclude Wagyu-sired steers because across-breed EPD adjustments are unavailable for the breed

Phenotype	Sire EPD	Pearson correlation coefficient	*P*-value
Initial body weight, kg	YW	0.02	0.74
Final body weight, kg	YW	0.03	0.64
Final body weight, kg	HCW	0.07	0.27
Average daily gain, kg/d	YW	0.17	0.009
Average daily gain, kg/d	ADG	0.07	0.27
Dry matter intake, kg/d	ADG	0.13	0.04
Gain-to-feed ratio, kg/kg	ADG	−0.04	0.51
Residual feed intake, kg	ADG	0.13	0.04
Hot carcass weight, kg	HCW	0.11	0.08
Ribeye area, cm^2^	REA	0.11	0.09
Backfat, mm	FAT	0.32	<0.001
Marbling score	MARB	0.14	0.03
Intramuscular fat, %	MARB	0.25	<0.001

YW, yearling weight; HCW, hot carcass weight; ADG, average daily gain; REA, ribeye area; FAT, fat thickness; MARB, marbling score.

**Table 5.** Pearson correlation coefficients between phenotypes of beef × Holstein steers (*n* = 259) and Igenity Beef molecular breeding values (MBV) of relevant traits

Phenotype	Igenity MBV	Pearson correlation coefficient	*P*-value
Initial body weight, kg	YW	0.22	<0.001
Final body weight, kg	HCW	0.22	<0.001
Average daily gain, kg/d	ADG	−0.08	0.19
Average daily gain, kg/d	RFI	0.16	0.01
Dry matter intake, kg/d	RFI	0.12	0.05
Gain-to-feed ratio, kg/kg	RFI	0.06	0.32
Residual feed intake, kg	RFI	0.07	0.28
Hot carcass weight, kg	HCW	0.22	<0.001
Ribeye area, cm^2^	REA	0.05	0.39
Backfat, mm	FAT	0.22	<0.001
Marbling score	MARB	0.21	<0.001
Intramuscular fat, %	MARB	0.27	<0.001
Warner-Bratzler shear force, kg	TEND	0.27	<0.001
Net profit	TI	0.21	<0.001

YW, yearling weight; ADG, average daily gain; RFI, residual feed intake; HCW, hot carcass weight; REA, ribeye area; FAT, fat thickness; MARB, marbling score; TEND, tenderness; TI, terminal index.

Regression coefficients of breed-adjusted sire EPD on performance phenotypes of beef × Holstein progeny are presented in Table 6. Regression coefficients of Igenity Beef MBV on performance phenotypes utilizing the full dataset are presented in Table 7 and those using the dataset excluding Charolais and Wagyu progeny are presented in Table 8. In models where EPD or MBV exist for a trait that directly corresponds to the phenotype measured, a regression coefficient of 1 indicates that the genetic effect size contributing to the phenotype is exactly as the breeding value predicted—e.g., if the phenotypes of carcass weight regressed on breed-adjusted sire EPD of carcass weight resulted in a regression coefficient of 1, every kg of carcass weight predicted by sire EPD was realized in the carcass weight phenotypes of beef × Holstein progeny. Likewise, when carcass weight is regressed on carcass weight MBV, a regression coefficient of 1 indicates that the steer’s genomic value for carcass weight was fully realized. Because Igenity Beef scores were converted to MBV, the effect size represented by the regression coefficient represents the predicted value in relation to the units of the actual phenotype, not the scale of 1 to 10. A regression coefficient less than 1 suggests that the breeding value overestimated the effect size and a regression coefficient greater than 1 suggests that the breeding value underestimated the effect size. In cases where breeding values were not available for the exact phenotypes measured, the regression coefficients inform how selecting upon breeding values for certain traits impact related phenotypes.

**Table 6.** Linear regression coefficients of breed-adjusted sire expected progeny differences (EPD) of relevant traits on beef × Holstein steer phenotypes (*n* = 248); data exclude Wagyu-sired steers because across-breed EPD adjustments are unavailable for the breed

Phenotype	Sire EPD	Model intercept ± SE	EPD regression coefficient ± SE	EPD *P*-value
Initial body weight, kg	YW	441 ± 20	0.70 ± 0.32	0.03
Final body weight, kg	HCW	681 ± 14	0.77 ± 0.33	0.02
Average daily gain, kg/day	ADG	1.62 ± 0.03	0.55 ± 0.22	0.01
Dry matter intake, kg/day	ADG	13.8 ± 0.2	5.7 ± 1.7	<0.001
Gain-to-feed ratio, kg/kg	ADG	0.120 ± 0.002	−0.01 ± 0.02	0.57
Residual feed intake, kg	ADG	−0.19 ± 0.18	2.40 ± 1.37	0.08
Hot carcass weight, kg	HCW	420 ± 8	0.80 ± 0.23	<0.001
Ribeye area, cm^2^	REA	83.9 ± 1.6	0.23 ± 0.20	0.25
Backfat, mm	FAT	13.2 ± 0.4	0.22 ± 0.15	0.14
Marbling score	MARB	5.1 ± 0.3	0.43 ± 0.13	0.001
Intramuscular fat, %	MARB	4.9 ± 0.5	1.0 ± 0.3	<0.001

YW, yearling weight; HCW, hot carcass weight; REA, ribeye area; FAT, fat thickness; MARB, marbling score.

**Table 7.** Linear regression coefficients of Igenity Beef molecular breeding values (MBV) of relevant traits on beef × Holstein steer phenotypes (*n* = 259)

Phenotype	Igenity MBV	Model intercept ± SE	MBV regression coefficient ± SE	MBV *P*-value
Initial body weight, kg	YW	442 ± 20	0.72 ± 0.31	0.02
Final body weight, kg	HCW	652 ± 17	0.74 ± 0.21	<0.001
Average daily gain, kg/day	ADG	1.64 ± 0.04	0.18 ± 0.31	0.58
Average daily gain, kg/day	RFI	1.55 ± 0.05	0.31 ± 0.12	0.009
Dry matter intake, kg/day	RFI	13.6 ± 0.3	2.0 ± 0.9	0.02
Gain-to-feed ratio, kg/kg	RFI	0.115 ± 0.003	0.006 ± 0.008	0.49
Residual feed intake, kg	RFI	−0.22 ± 0.27	0.70 ± 0.68	0.30
Hot carcass weight, kg	HCW	404 ± 11	0.52 ± 0.15	<0.001
Ribeye area, cm^2^	REA	83.6 ± 2.5	0.14 ± 0.17	0.40
Backfat, mm	FAT	12.0 ± 0.7	0.22 ± 0.12	0.07
Marbling score	MARB	4.65 ± 0.35	0.42 ± 0.12	<0.001
Intramuscular fat, %	MARB	4.01 ± 0.60	0.89 ± 0.26	<0.001
Warner-Bratzler shear force, kg	TEND	4.63 ± 0.21	1.40 ± 0.32	<0.001
Net profit, USD	TI	327 ± 124	43 ± 20	0.03

YW, yearling weight; HCW, hot carcass weight; ADG, average daily gain; RFI, residual feed intake; REA, ribeye area; FAT, fat thickness, MARB, marbling score; TEND, tenderness; TI, terminal index.

**Table 8.** Linear regression coefficients of Igenity Beef molecular breeding values (MBV) of relevant traits on beef × Holstein steer phenotypes (*n* = 157); data exclude MBV and phenotypes from steers sired by Charolais and Wagyu bulls because the breeds were not used in the development of the Igenity Beef genomic test

Phenotype	Igenity MBV	Model intercept ± SE	MBV regression coefficient ± SE	MBV *P*-value
Initial body weight, kg	YW	392 ± 25	1.97 ± 0.49	<0.001
Final body weight, kg	HCW	651 ± 22	0.70 ± 0.30	0.02
Average daily gain, kg/d	ADG	1.57 ± 0.07	0.65 ± 0.53	0.22
Average daily gain, kg/d	RFI	1.52 ± 0.05	0.37 ± 0.14	0.008
Dry matter intake, kg/d	RFI	13.4 ± 0.5	2.14 ± 1.07	0.05
Gain-to-feed ratio, kg/kg	RFI	0.111 ± 0.004	0.013 ± 0.010	0.22
Residual feed intake, kg	RFI	−0.07 ± 0.31	0.23 ± 0.83	0.78
Hot carcass weight, kg	HCW	402 ± 15	0.54 ± 0.22	0.01
Ribeye area, cm^2^	REA	82.7 ± 3.1	0.20 ± 0.21	0.34
Backfat, mm	FAT	12.7 ± 1.0	0.13 ± 0.18	0.47
Marbling score	MARB	4.34 ± 0.37	0.67 ± 0.15	<0.001
Intramuscular fat, %	MARB	4.03 ± 0.72	0.83 ± 0.35	0.02
Warner-Bratzler shear force, kg	TEND	4.61 ± 0.26	1.51 ± 0.37	<0.001
Net profit, USD	TI	173 ± 186	68 ± 31	0.03

YW, yearling weight; HCW, hot carcass weight; ADG, average daily gain; RFI, residual feed intake; REA, ribeye area; FAT, fat thickness; MARB, marbling score; TEND, tenderness; TI, terminal index.

### Growth traits

**Figure 1.**
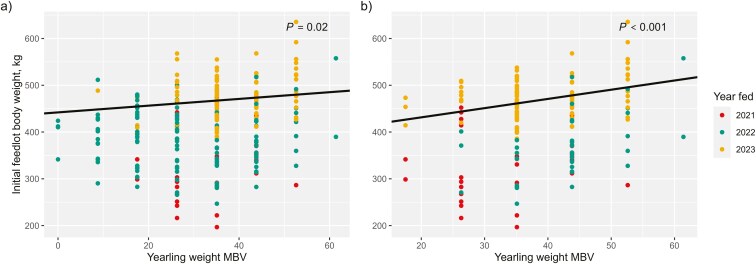
Initial feedlot body weight phenotypes of beef × Holstein steers regressed on molecular breeding value (MBV) of yearling weight from a) the complete data set (*n* = 259) and b) the data excluding Charolais and Wagyu-sired steers (*n* = 157). Point color denotes the year a steer was fed, which was a significant fixed effect included in models.

Correlations between sire EPD of YW and initial body weight and between sire EPD of HCW and final body weight were not different from 0 (Table 4). However, when accounting for model effects, every additional kg of YW in a sire’s EPD resulted in progeny 0.70 kg heavier at feedlot arrival (*P *= 0.03; Table 6); likewise, every kg of HCW in a sire’s EPD was associated with beef × Holstein steers that were 0.77 kg heavier at slaughter (*P *= 0.01; Table 6). The lack of correlation may be because initial and final body weights were not adjusted for steer age because birthdates were not available for all animals. The addition of year on feed and herd of origin in the regression models likely captured some of the age differences between contemporary groups, thus a relationship was detected. Igenity MBV of YW were positively correlated with the body weight of beef × Holstein steers at arrival to the feedlot (initial body weight) and MBV of HCW were positively correlated with body weight leaving the feedlot (final body weight; Table 5); each kg of YW in a steer’s MBV was associated with 0.72 kg greater body weight at feedlot arrival (*P* < 0.001; Table 7; Figure 1a). Each kg of HCW MBV was associated with 0.74 kg greater body weight at departure (*P *< 0.05; Table 7); the effect size in the data excluding Charolais and Wagyu progeny was nearly identical (Table 8). The effect size of YW MBV on initial body weight was increased to 1.97 kg following the removal of steers sired by breeds that were not supported by the Igenity Beef test (*P *< 0.01; Table 8; Figure 1b). The increase in effect size following the removal of Charolais and Wagyu-sired steers could be due to Igenity Beef more accurately ranking progeny of supported breeds by genotype for YW. However, the sample size of genotyped steers was reduced by 40% when the progeny of unsupported breeds were removed from the data. It is likely that fewer phenotypic observations attributed to effect size differences in the model of initial body weight.

**Figure 2.**
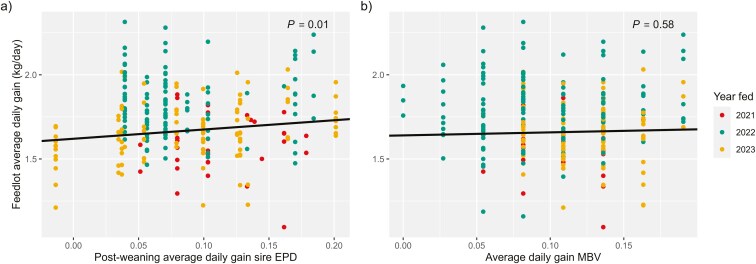
Feedlot average daily gain phenotypes of beef × Holstein steers regressed on a) derived breed-adjusted sire expected progeny differences (EPD) of average daily gain (*n* = 248) and b) molecular breeding value (MBV) of average daily gain (*n* = 259). Point color denotes the year a steer was fed, which was a significant fixed effect included in models.

The MBV of ADG were not correlated with phenotypic ADG of steers fed and did not explain variation in ADG between the steers tested (*P* > 0.05; Table 7; Figure 2a), even when removing phenotypes from progeny of Wagyu and Charolais sires (*P* > 0.05; Table 8). The derived breed-adjusted sire EPD of postweaning ADG were not correlated with phenotypic ADG of beef × Holstein steers (Table 4). However, for every additional kg of postweaning ADG in a sire’s EPD, his beef × Holstein progeny had 0.55 kg greater ADG over the feeding period (*P *= 0.01; Table 6; Figure 2b).

Where MBV of ADG could be advantageous is for those purchasing feeder calves without information on genetic background. One of the features specifically marketed for genomic testing terminal animals is a derived expected days on feed based on ADG MBV so that animals can be grouped and managed based on expected growth (Neogen, 2021). However, MBV of ADG were not associated with phenotypic ADG of the beef × Holstein steers in this study, while breed-adjusted sire EPD of postweaning ADG EPD were (Figure 2). Postweaning ADG is not a trait common across beef-breed genetic evaluations, though some form of postweaning ADG EPD are available for the breeds Angus, Simmental, and Red Angus (RAAA, 2021; AAA, 2024; AAA, 2024; ASA, 2024). As demonstrated in this study, the EPD of weaning weight and YW, which are more ubiquitous across breeds, are sufficient to derive an EPD that can adequately predict phenotypic ADG. Thus, when the sire is known, a derived sire EPD of postweaning ADG is a parameter that can inform beef × dairy calf buyers about expected feedlot gains.

Sire EPD of postweaning ADG were weakly positively correlated with phenotypic DMI of growing beef × Holstein steers (*r* = 0.13; Table 4). For every additional kg of ADG in a sire’s EPD, his beef × Holstein progeny ate 5.7 kg more dry matter, daily (*P *< 0.01; Table 6). The direction of this relationship is logical as feed intake is positively genetically and phenotypically correlated with ADG (Crowley et al., 2010; Rolfe et al., 2011; Mao et al., 2013; Ceacero et al., 2016). Sire EPD of ADG were not a predictor of phenotypic feed efficiency when measured as gain-to-feed ratio (*P *> 0.05), though for every kg of ADG in a sire’s EPD beef × Holsteins tended to have 2.40 kg greater phenotypic RFI (*P *= 0.08). These results are contradictory to previous works in full-blood beef cattle which estimated that ADG is positively genetically and phenotypically correlated with gain-to-feed while ADG and RFI are not phenotypically or genetically correlated (Crowley et al., 2010; Rolfe et al., 2011; Mao et al., 2013). This disparity may be due to the EPD of postweaning gain not being equivalent to feedlot ADG, which is when feed efficiency was measured in the beef × Holstein steers in this study. Gain-to-feed ratio is the efficiency metric more commonly considered by cattle feeders; even if sire EPD of ADG may impact phenotypic RFI of beef × Holstein steers, if it does not negatively influence gain-to-feed ratio it is unlikely to have a significant impact on economic return.

**Figure 3.**
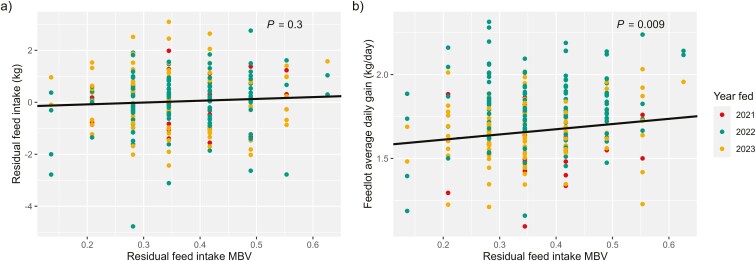
Molecular breeding value (MBV) of residual feed intake a) did not explain variation in phenotypic residual feed intake of beef × Holstein steers, b) though residual feed intake MBV was associated with greater feedlot average daily gain. Point color denotes the year a steer was fed, which was a significant fixed effect included in models.

In both the full and reduced data, there was no relationship between RFI MBV and gain-to-feed ratio or phenotypic RFI (*P *> 0.05; Tables 7 and 8; Figure 3a). Phenotypes of ADG were positively correlated with MBV of RFI (*r* = 0.16; Table 5). For each additional kg of RFI MBV, beef × Holstein steers gained 0.31 kg more per day (*P *< 0.01; Table 7; Figure 3b). The effect of RFI MBV on ADG was similar when Charolais and Wagyu-sired progeny were removed from the data (Table 8). Phenotypic DMI was weakly, positively correlated with RFI MBV (*r* = 0.12; Table 5) and, for each kg of RFI predicted by MBV, phenotypic DMI was increased in beef × Holstein steers by 2 kg (*P *= 0.02; Table 7). A relationship between RFI MBV and phenotypic DMI of similar magnitude existed when Charolais and Wagyu-sired progeny were removed from the data (Table 8).

Residual feed intake is indicative of the amount of additional feed an animal needs to consume to maintain the same rate of gain as their contemporaries, thus, in theory, should be independent of gain (Koch et al., 1963). The greater the RFI, the less feed efficient an animal is in comparison with their contemporaries. In this experiment, the beef × Holstein steers with greater ADG had greater MBV of RFI indicating that RFI MBV were not independent of gain. Previous studies that have estimated genetic parameters of RFI and ADG in growing cattle suggest that the 2 traits are not genetically or phenotypically correlated (Crowley et al., 2010; Rolfe et al., 2011; Mao et al., 2013). There was a weak positive correlation between DMI phenotypes and RFI MBV and RFI MBV predicted phenotypic DMI. This is consistent with existing literature that has estimated positive genetic and phenotypic correlations between RFI and DMI (Crowley et al., 2010; Rolfe et al., 2011; Mao et al., 2013). Conversely, RFI MBV was not associated with gain-to-feed ratio or phenotypic RFI regardless of breed inclusion in the data (*P* > 0.05; Figure 3a). The lack of relationship between RFI MBV and feed efficiency phenotypes is contradictory to the literature on RFI in growing cattle where RFI has been found to be negatively genetically and phenotypically correlated with gain-to-feed ratio (Crowley et al., 2010; Rolfe et al., 2011; Mao et al., 2013). The results presented here suggest that RFI MBV derived by Igenity Beef does not accurately reflect the phenotypic RFI of beef × Holstein steers, though it does predict DMI. Further, the antagonistic relationship between RFI MBV and phenotypic ADG suggests Igenity MBV of RFI may not translate to true RFI in beef × Holstein steers (Figure 3). The dairy genetic influence of the cattle fed in this study may have impacted the predictive ability of the genomic test for RFI. Feed intake phenotypes of cattle can be expensive and more challenging to collect than growth and carcass phenotypes. There are not published accuracies associated with Igenity MBV, but it is possible that the amount of feed intake phenotypes in the reference population of Igenity Beef resulted in the accuracy of RFI MBV being smaller than for the other Igenity MBV evaluated in this study. A relationship between RFI MBV and phenotypic RFI may have been detected with a larger validation population of beef × Holstein cattle.

Molecular breeding values derived by the Igenity beef genomic test has the advantage over breed-adjusted sire EPD of providing an estimate for an additional trait, RFI. However, RFI MBV were associated with phenotypic ADG rather than feed efficiency measures. Furthermore, ADG MBV were not associated with phenotypic ADG of the beef × Holstein steers in this study, while steers sired by bulls with greater EPD of ADG had greater phenotypic ADG (Figure 2). Both breed-adjusted sire EPD and MBV of YW and HCW corresponded with initial and final feedlot body weights, respectively, of beef × Holstein steers in this study. While accuracies were not available for MBV, the average accuracies of weaning weight and YW EPD used to derive ADG EPD were moderate (0.71 and 0.66, respectively). Results suggest that MBV from Igenity Beef provides no better predictions of feedlot growth of beef × Holstien steers than across-breed sire EPD. Sire EPD explained more variation in ADG phenotypes than MBV, which could be due to EPD having greater accuracy by including progeny phenotypes in their derivation.

### Carcass traits

**Figure 4.**
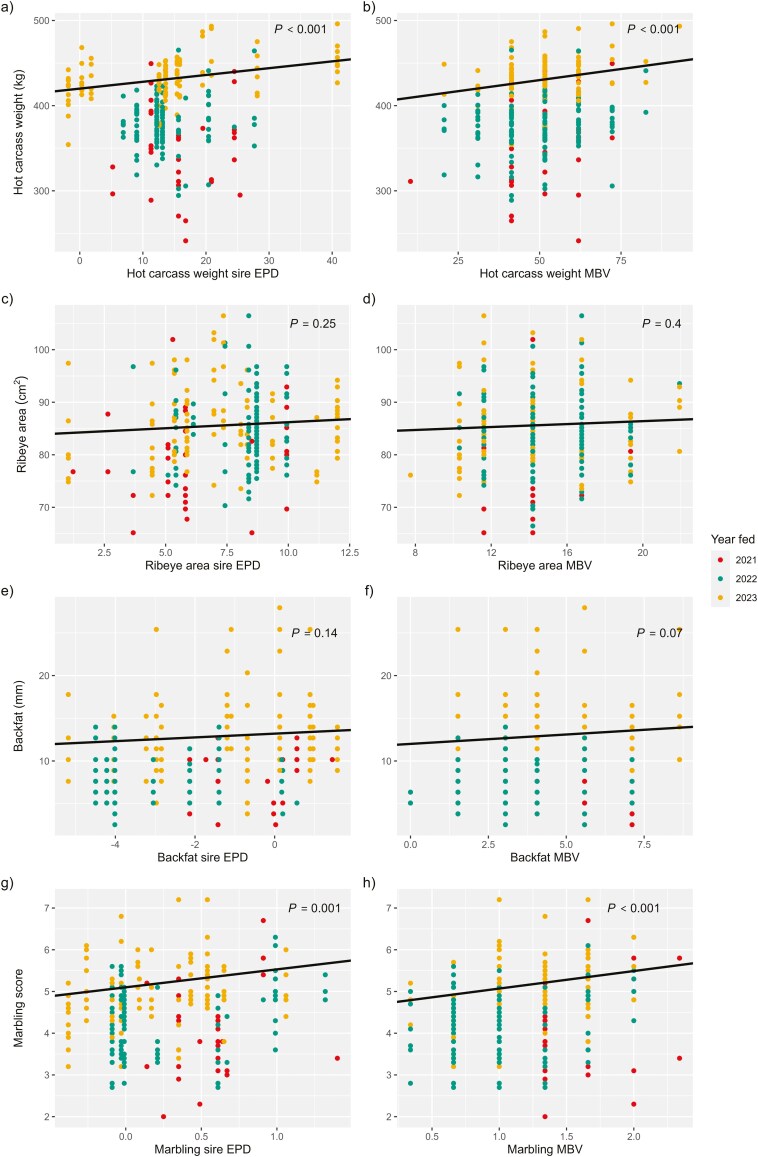
Carcass phenotypes of beef × Holstein steers regressed on associated breed-adjusted sire expected progeny differences (EPD) and molecular breeding values (MBV). Phenotypes include a) hot carcass weight (HCW) regressed on HCW EPD and b) HCW MBV, c) ribeye area (REA) regressed on REA EPD and d) REA MBV, e) backfat regressed on backfat EPD and f) backfat MBV, and g) marbling score regressed on marbling EPD and h) marbling MBV. Point color denotes the year a steer was fed, which was a significant fixed effect included in models.

Phenotypic carcass weight was positively correlated with HCW MBV (*r* = 0.22; Table 5). For each kg of HCW predicted by MBV, beef × Holstein carcasses were 0.52 kg heavier (*P* < 0.01; Table 7; Figure 4a). When the phenotypes of Wagyu and Charolais-sired steers were removed from the data, the regression coefficient was nearly identical (Table 8). Though the weak, positive correlation between breed-adjusted sire EPD of HCW and HCW phenotype only tended to differ from 0 (*r* = 0.11; Table 4), the regression coefficient of HCW sire EPD on HCW was greater than that of HCW MBV (0.8 kg; *P* < 0.01; Table 6; Figure 4b). Breed-adjusted sire EPD of HCW were only moderately correlated (*r* = 0.33) with MBV of HCW; the correlation was less than correlations between EPD and MBV for all other traits with both breeding values available, implying that sires were ranked by HCW EPD differently than their beef × Holstein progeny ranked for HCW MBV (Table 3). Both MBV derived for HCW by Igenity Beef and breed-adjusted sire EPD of HCW were able to accurately predict phenotypic HCW of beef × Holstein steers. Effect size differences suggest that phenotypic differences in HCW were better explained by sire EPD than MBV. The genetic influence of the dairy dam may have impacted the genomic test’s ability to accurately predict HCW MBV of beef × dairy steers.

A weak, positive correlation between breed-adjusted sire EPD of REA and phenotypes of REA tended to differ from 0 (*r* = 0.11; Table 4). Ribeye area phenotypes were not correlated with MBV of REA and, when sire EPD or MBV of REA were regressed on phenotypic REA, the breeding values did not explain any variation in the phenotype (*P* > 0.05; Figure 4c and 4d). The same was true of the data that excluded MBV of Charolais and Wagyu-sired progeny. More phenotypes from REA from beef × Holstein steers may have allowed for a relationship to be detected. However, relationships between carcass weight phenotype and breeding values, both sire EPD and MBV, of HCW were detected from this data. We would expect to detect a similar relationship between REA phenotype and breeding values as REA has a similar heritability to HCW (Utrera and Van Vleck, 2004). Further, the correlation between sire EPD and MBV of REA was nearly twice that of the EPD and MBV of HCW, suggesting that both types of breeding value were ranking cattle more similarly for REA than for HCW. It should be noted that the average accuracy of REA sire EPD was the smallest among the traits evaluated (0.51) but did not differ greatly from the average accuracies of sire EPD of HCW or marbling (0.55 and 0.53, respectively).

It is possible that the Holstein genetic influence masks additive genetic effects that contribute to REA in beef-breed populations. A potential reason that MBV did not accurately rank beef × Holstein progeny by REA could be related to marker selection on the genomic panel. Even if the same genes and genomic regions modulate REA across all cattle breeds, allelic differences that occur between haplotype makers could be unique to the Holstein breed and impact REA. The same would be true in relation to sire EPD, as genomic markers are utilized in beef cattle genetic evaluations. Little research has been performed to examine genetic variation or identify genomic regions associated with REA in the Holstein breed because of their primary function as dairy cattle. One study utilized repeated measures of longissimus muscle area by ultrasound from 202 Holstein cows (Sloniewski et al., 2004). Authors concluded that additive genetic variance contributed to most of the variation in ultrasound longissimus muscle area of Holstein cows; heritability was estimated as 0.69 (Sloniewski et al., 2004). The genetic variation that Holstein dams contribute to REA in beef × Holstein progeny warrants further exploration. Phenotypes of REA from beef × Holstein progeny could be used to improve MBV estimates and marker selection for the trait.

Backfat thickness was positively correlated with sire EPD of FAT and MBV of FAT. The relationship between sire EPD of FAT and phenotypic backfat thickness was somewhat stronger (*r* = 0.32; Table 4) than the relationship between MBV of FAT and backfat thickness (*r* = 0.22; Table 5). For every mm of backfat predicted by MBV, beef × Holstein carcasses tended to have 0.22 mm greater backfat, about half of the expected effect (*P *= 0.07; Table 7; Figure 4e). Despite the strength of the correlation between sire EPD of FAT and fat thickness, sire EPD of FAT did not explain variation in backfat thickness when linearly modeled (Figure 4f). In the data that excluded Wagyu and Charolais-sired progeny, there was no relationship between FAT MBV and carcass backfat.

Like with the ribeye area, it is possible that the dairy genetics of the beef × Holstein steers masked genetic differences in backfat. It has been demonstrated that carcasses of Holstein steers have less backfat than native breeds of cattle (Boykin et al., 2017). A number of older studies observed that backfat thickness was similar between dairy breeds and beef × dairy crosses of the same dairy breed and sex (Forrest, 1977, 1980, 1981; Baker et al., 1984). More recent work observed that carcasses from Jersey steers had 5.1 mm less backfat than those from Angus × Jersey steers while the backfat of carcasses from SimAngus × Jersey steers and Red Wagyu × Jersey steers did not differ from the Jersey carcasses or Angus × Jersey carcasses (Jaborek et al., 2019). Another study reported that full-blood beef-breed carcasses had, on average, 2 mm more backfat than beef × dairy carcasses and beef × dairy carcasses exceeded Holstein carcasses in backfat by 1.9 mm (Foraker et al., 2022b). The literature suggests that the backfat of beef × dairy carcasses is often more similar to that of dairy-breed carcasses or intermediate between dairy and beef-breed carcasses. It is likely that the small effect size MBV of backfat tended to have on phenotypic backfat is due to the dairy genetic influence in the beef × Holstein steers. Regardless, breeding values of backfat are unlikely to be economically relevant for those selling beef × dairy cattle on the grid as discounts are applied for too much backfat, but not for too little. Greater backfat thickness contributes to greater USDA Yield Grades (USDA, 2017), and accordingly, Foraker et al. (2022b) reported a smaller proportion of both beef × dairy and Holstein carcasses receiving Yield Grade discounts when compared with native beef carcasses.

Marbling score and IMF percentage were positively correlated with marbling MBV (*r* = 0.21 and *r* = 0.27, respectively; Table 5) and breed-adjusted sire EPD (*r* = 0.14 and *r* = 0.25, respectively; Table 4). For each point of marbling score predicted by sire EPD, carcasses had a 0.43 point increase in marbling score (Figure 4g) and the longissimus muscle contained 1.0% more IMF (*P *< 0.01; Table 6). Similarly, for each point of marbling score predicted by MBV, carcasses had a 0.42 point increase in marbling score (Figure 4h) and the longissimus muscle contained 0.89% more IMF (*P *< 0.01; Table 7). Marbling MBV were similar to sire EPD as direct predictors of marbling score; when Charolais and Wagyu-sired progeny were removed from the data, MBV had a greater marbling score effect at 0.67 points (*P *< 0.01; Table 8). In the data that excluded Charolais and Wagyu-sired beef × Holstein steers, marbling MBV predicted IMF similarly to MBV in the complete data set (0.83%; *P = *0.02; Table 8). Marbling score is the sole parameter evaluated for USDA Quality Grade in carcasses from steers under 30 months of age (USDA, 2017), which makes it an important factor in carcass valuation on the grid. Either marbling score MBV or sire EPD could be used by cattle feeders to determine how to market fed beef × dairy cattle. The larger effect size of MBV when Charolais and Wagyu-sired progeny were removed from the data suggests that MBV may have a small advantage over sire EPD when cattle are sired only by breeds supported by the genomic test. In any case, producers can market cattle that have moderate to high marbling breeding values on the grid to achieve quality premiums while beef × Holstein cattle with low marbling MBV may be more profitable when marketed on a live basis.

Tenderness MBV were positively correlated with Warner-Bratzler shear force tenderness measured in the longissimus muscle (*r* = 0.27; Table 5). For every kg of force predicted to be required to shear the longissimus muscle, 1.40 kg of force was required (*P *< 0.01; Table 7). The coefficient increased to 1.51 kg when Wagyu and Charolais progeny were excluded from the data (Table 8). Regardless of sire breed, the tenderness MBV from Igenity Beef was able to predict how beef × Holstein carcasses would rank in tenderness. Tenderness was one of the first traits commercially marketed for genomic prediction in beef cattle; the original tests used only 3 single nucleotide polymorphisms as markers for the trait, which were independently validated as strongly associated with tenderness phenotypes (Van Eenennaam et al., 2007). The results presented here support previous validation of tenderness MBV and further validate that tenderness MBV can be used for beef × Holstein progeny, even when sired by beef breeds that were not utilized in genomic test development.

### Profitability

**Figure 5.**
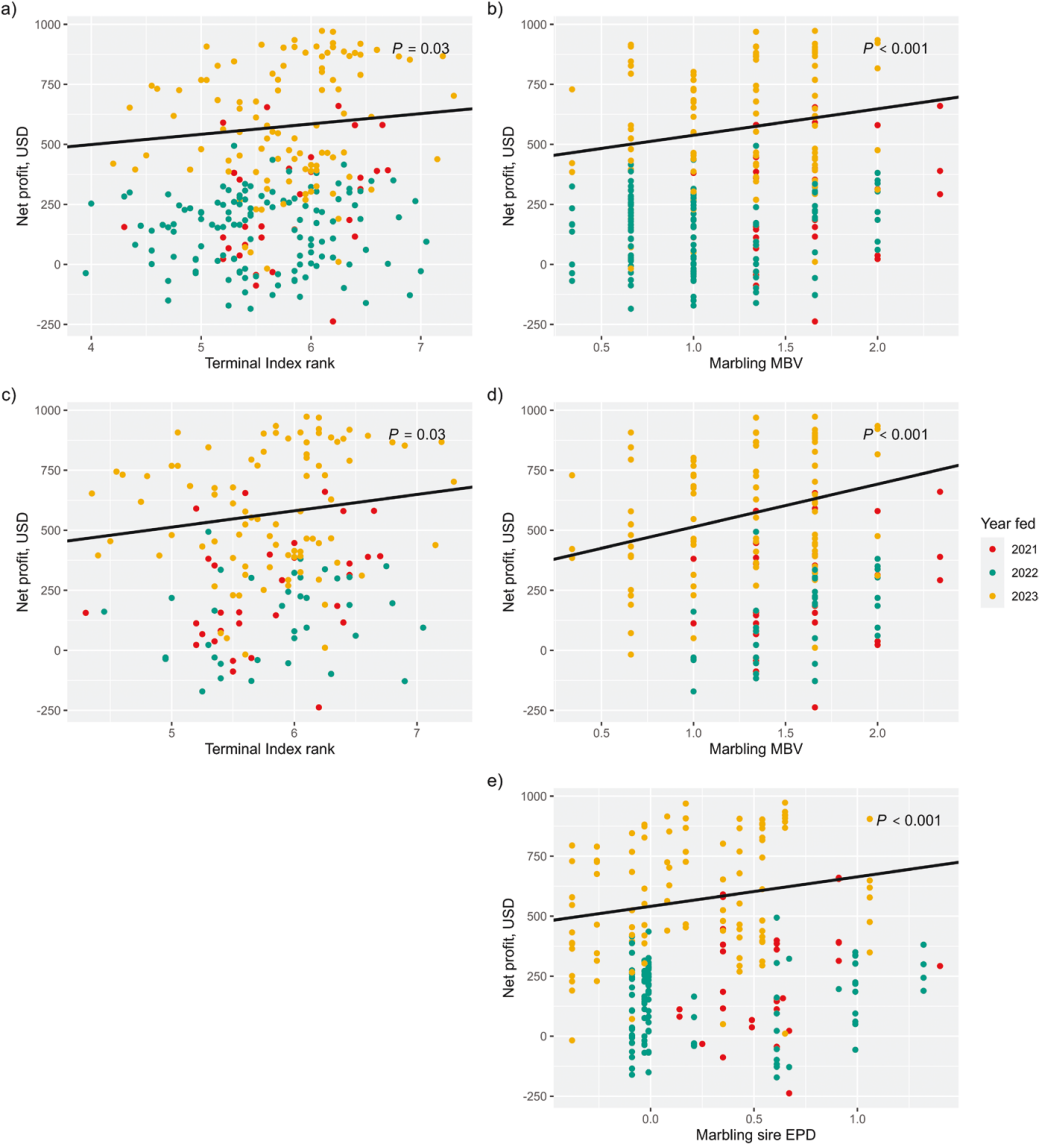
Feedlot net profit of beef × Holstein steers regressed on a) Igenity Terminal Index ranking and b) marbling molecular breeding values (MBV) from the complete data set (*n* = 259); c) net profit regressed on Igenity Terminal Index ranking and d) marbling MBV from the data excluding Charolais and Wagyu-sired steers (*n* = 157); e) net profit regressed on sire expected progeny differences (EPD) of marbling (*n* = 248). Point color denotes the year a steer was fed, which was a significant fixed effect included in models.

Terminal index ranking (from 1 to 10) generated by Igenity Beef was positively correlated with the net profit of beef × Holstein steers fed in this study (*r* = 0.20; Table 5). Net profit increased as a steer’s ranking on the TI increased; for each additional point in TI ranking net profit increased by $43 USD (*P *= 0.03; Table 7; Figure 5a). The effect was greater ($68) when Charolais and Wagyu-sired cattle were removed from the data (*P *= 0.03; Table 8; Figure 5c).

Previous research has suggested that profit from differential pricing and management of fed beef-breed cattle based on MBV is less than the cost of genomic testing, which at the time was approximately $40 per animal (Thompson et al., 2014, 2016). Selectively marketing finished cattle by live weight, dressed weight, or on the grid based on MBV was estimated to gross between $1 and $13 of additional profit per head (Thompson et al., 2016). However, using MBV to decide the purchasing price of feeder cattle and to adjust management based on MBV was estimated to result in up to $38 of gross profit (Thompson et al., 2014). Since then, the cost of Igenity Beef has been reduced to $30, and 2 more affordable genomic tests were released: Igenity Feeder ($15), and Igenity BeefxDairy ($17; Neogen, 2021, 2023a, 2023b). Based on the models of Thompson et al. (2014), currently, there could be net profit potential from utilizing genotypes of beef-breed feeder cattle to determine buying price and feedlot management groups. In this study, MBV of ADG, which is the breeding value used to estimate days on feed for precision management of steer groups, was not an accurate predictor of phenotypic ADG in beef × Holstein steers. This negates one of the major economic benefits of genotyping feeder cattle.

Based on the results of this study, most of the value predicted by the TI is likely attributed to HCW and marbling MBV. The TI puts positive weights on the MBV of HCW (40%), REA (10%), marbling (20%), and tenderness (5%) and negative weight on FAT (-10%) and RFI (−15%) MBV (Neogen, 2023a). Among those traits, HCW, marbling, and tenderness MBV were the only MBV that significantly explained variation in the corresponding phenotypes of beef × Holstein steers. The steers in this study were sold on the grid, where tenderness does not contribute to carcass value while marbling does. To validate these findings, TI rank was replaced with marbling MBV by Igenity Beef and regressed on net profit using the same model effects. The regression coefficient was $110 ± $30 (*P* < 0.001; Figure 5b). When the same model was run with the data excluding Charolais and Wagyu-sired progeny, an even greater genetic response was observed; for every additional point in marbling score MBV, beef × Holstein steers net an additional $178 (±$44; *P *< 0.001; Figure 5d). The stronger relationship between marbling score MBV and net profit further supports that the MBV of the other traits included in the TI are not contributing to better estimates of net profit. In fact, by putting negative weight on RFI MBV, which was not associated with feed efficiency in this study but was positively associated with ADG, the index puts more value on beef × dairy steers with less phenotypic ADG.

Terminal selection indexes are available for many of the beef breeds used to sire the beef × Holstein progeny in this study. However, each index uses different economic weights for different traits and are expressed relative to a base of purebred cows that varies depending on breed characteristics such as a high level of marbling in Angus or growth in Charolais; these make it challenging to translate the index values between breeds. One advantage to utilizing a multi-breed genomic test is having an economic index value that is comparable across breed backgrounds. Because marbling MBV was strongly associated with feedlot net profit, breed-adjusted sire EPD were also regressed on net profit using the same model effects from the other EPD models. The regression coefficient was $123 ± $34 (*P* < 0.001; Figure 5e), indicating that sire marbling EPD was similarly capable to marbling MBV in predicting net profit. Like with marbling score, the genetic effect of marbling MBV was associated with greater net profit than sire EPD when Charolais and Wagyu-sired progeny were excluded from the data.

Sire EPD and MBV of marbling were stronger indicators of the net profit of beef × Holstein steers sold on the grid than the Igenity TI. Further, ADG MBV did not accurately rank beef × Holstein steers by phenotypic ADG, suggesting that precision management of beef × dairy steers by ADG MBV would not result in anticipated economic advantages. It is important to note that the economic information used here only reflects a few points of time in a fluctuating market. In this study, steers were not precision managed based on breeding values, nor were they marketed differently based on TI ranking, thus we cannot make strong conclusions on the anticipated profit margins of implementing genomic testing of terminal beef × Holstein steers. Further economic analyses are required to verify where genomic testing of fed beef × dairy progeny could add similar value, provided that genomic testing results can be obtained prior to cattle being purchased by the feeder.

### Application

Molecular breeding values derived from the Igenity Beef genomic test were similar to breed-adjusted sire EPD in predicting phenotypes of corresponding growth and carcass traits in beef × Holstein progeny. Both MBV and sire EPD were poor predictors of ribeye area and backfat thickness of beef × Holstein carcasses, while sire EPD of YW was a better predictor of ADG than MBV of ADG. Molecular breeding values of marbling were a stronger predictor of marbling score and IMF percentage of beef × Holstein carcasses than sire EPD of marbling. The genomic test also had the advantage of accurately ranking cattle based on carcass tenderness as an additional indicator of quality, though currently, no carcass grids pay premiums on tenderness. The TI value generated by Igenity Beef was associated with greater feedlot profitability; however, marbling ranking derived from MBV had a stronger relationship with profitability because MBV of other traits included in the index were not associated with the phenotypes of beef × Holstein steers. For traits like REA, where neither sire EPD nor MBV were predictors of REA phenotype in beef × Holstein progeny, estimates of breeding values may be improved if derived using phenotypes of beef × Holstein progeny.

Though Igenity Beef is not marketed for the progeny of Holstein, Charolais, and Wagyu breeds, it could predict the phenotypic performance of progeny of Holstein dams for the same traits sire EPD could. Likewise, the removal of Charolais and Wagyu progeny from the data did not improve the predictive ability for traits that MBV were poor predictors for, such as ADG, REA, and backfat thickness. However, marbling MBV had a slightly greater genetic effect than sire EPD of marbling on marbling score phenotypes and feedlot net profit when the progeny of unsupported breeds were removed.

A genomic panel specifically for beef × dairy progeny, Igenity BeefxDairy, was released following the initiation of this experiment (Neogen, 2023b). The BeefxDairy genomic test provides customers with MBV ranking for ADG, HCW, and marbling as well as ranking on the same TI used by Igenity Beef, and is labeled to support Wagyu-sired progeny (Neogen, 2023b). The published MBV associated with the ranking of the provided traits are the same values that were used to analyze these data—i.e., the published Igenity Beef MPD multiplied by 2 to convert the value to represent the terminal animal, rather than its progeny (Neogen, 2023a, 2023b). It is unclear if phenotypes from beef × dairy progeny were used to derive MBV and select markers for the beef × dairy test; the identical MBV between the tests suggests that the same markers and phenotypes used to develop Igenity Beef were used to derive Igenity BeefxDairy predictions. In that case, ADG ranking is unlikely to reflect actual phenotypes of beef × dairy progeny, as seen in this study. Likewise, the TI ranking may be more accurate by excluding traits where MBV are not associated with the phenotypes of beef × Holstein progeny.

Beef × dairy production has great potential for traceability because of the record-keeping associated with dairy production. As sire EPD were demonstrated to have some predictive ability of growth and carcass traits of beef × dairy progeny, calf buyers could take advantage of sire identification from herd records to assign value to calf groups purchased from dairies. Where genomic information could hold value is to buyers further down the supply chain. For example, buyers purchasing weaned beef × dairy progeny from a wet calf grower that does not have sire information available could utilize genomic ranking to inform purchasing price. The commercial genomic test, Igenity Beef, has the potential to be utilized in beef × dairy calf valuation, though currently, breed-adjusted sire EPD can provide nearly identical information.

## Abbreviations

ADG: average daily gain
DMI: dry matter intake
EPD: expected progeny differences
FAT: backfat thickness
HCW: hot carcass weight
IMF: intramuscular fat
LEC: Livestock Evaluation Center
MBV: molecular breeding value
MPD: molecular progeny differences
REA: ribeye area
RFI: residual feed intake
TI: terminal index
YW: yearling weight
